# TBHQ Alleviated Endoplasmic Reticulum Stress-Apoptosis and Oxidative Stress by PERK-Nrf2 Crosstalk in Methamphetamine-Induced Chronic Pulmonary Toxicity

**DOI:** 10.1155/2017/4310475

**Published:** 2017-02-20

**Authors:** Yun Wang, Yu-Han Gu, Ming Liu, Yang Bai, Li-Ye Liang, Huai-Liang Wang

**Affiliations:** ^1^Department of Clinical Pharmacology, School of Pharmacy, China Medical University, Shenyang 110122, China; ^2^Department of Drug Control, China Criminal Police University, Shenyang 110035, China; ^3^National Key Subject, Institute of Respiratory Diseases, China Medical University, Shenyang 110001, China

## Abstract

Methamphetamine (MA) leads to cardiac and pulmonary toxicity expressed as increases in inflammatory responses and oxidative stress. However, some interactions may exist between oxidative stress and endoplasmic reticulum stress (ERS). The current study is designed to investigate if both oxidative stress and ERS are involved in MA-induced chronic pulmonary toxicity and if antioxidant tertiary butylhydroquinone (TBHQ) alleviated ERS-apoptosis and oxidative stress by PERK-Nrf2 crosstalk. In this study, the rats were randomly divided into control group, MA-treated group (MA), and MA plus TBHQ-treated group (MA + TBHQ). Chronic exposure to MA resulted in slower growth of weight and pulmonary toxicity of the rats by increasing the pulmonary arterial pressure, promoting the hypertrophy of right ventricle and the remodeling of pulmonary arteries. MA inhibited the Nrf2-mediated antioxidative stress by downregulation of Nrf2, GCS, and HO-1 and upregulation of SOD2. MA increased GRP78 to induce ERS. Overexpression and phosphorylation of PERK rapidly phosphorylated eIF2*α*, increased ATF4, CHOP, bax, caspase 3, and caspase 12, and decreased bcl-2. These changes can be reversed by antioxidant TBHQ through upregulating expression of Nrf2. The above results indicated that TBHQ can alleviate MA-induced oxidative stress which can accelerate ERS to initiate PERK-dependent apoptosis and that PERK/Nrf2 is likely to be the key crosstalk between oxidative stress and ERS in MA-induced chronic pulmonary toxicity.

## 1. Introduction

Methamphetamine (MA) abuse is a major public health concern [1]. For methamphetamine abusers, one of the important death causes is cardiovascular complications including hypertension, aortic dissection, acute coronary syndromes, methamphetamine-associated cardiomyopathy, and pulmonary arterial hypertension (PAH) [2]. Chronic methamphetamine abuse is an increasingly common cause of PAH [3]. Increasing evidences suggested that methamphetamine administration leads to cardiac and pulmonary toxicity expressed as increases in inflammatory responses and oxidative stress [3–5].

Oxidative stress is a susceptible factor for endoplasmic reticulum stress (ERS) [6]. ERS has recently been paid more and more attention. ERS may initiate the unfolded protein response (UPR) to restore proteostasis or to lead to apoptosis [7]. Recent reports have suggested that the UPR signaling switches from prosurvival to proapoptosis through three ER transmembrane sensors: protein kinase-like ER kinase (PERK), activating transcription factor 6 (ATF6), and inositol-requiring enzyme-1*α* (IRE1) [8]. Remarkable evidence has indicated that ERS is accelerated by accumulation of unfolded/misfolded proteins after endoplasmic reticulum environment disturbance, triggered by a variety of physiological and pathological factors, such as nutrient deprivation, altered glycosylation, calcium depletion, DNA damage, energy disturbance, and oxidative stress [9]. In the condition of ERS, the overexpression of PERK will phosphorylate eIF2*α*, and then the p-eIF2*α* will transcribe some transcription factors, such as activating transcription factor 4 (ATF-4), C/EBP homologous protein (CHOP), and caspase 12 [10], which affect the apoptosis of cells or tissues through PERK-dependent pathway.

Activation of PERK signal can induce conformational change of nuclear factor E2-related factor 2 (Nrf2), which triggers the dissociation of Kelch-like ECH-associated protein 1- (Keap1-) Nrf2 complex to adjust the oxidation-redox condition of cells [11]. Nrf2 can enhance the transcription of cytoprotective genes during oxidative stress [12]. In addition, it has been reported that Nrf2 is involved in increasing the levels of endogenous antioxidants, attenuating apoptosis, and increasing mitochondrial biogenesis [13–15]. According to the above, it is prompted that there are some interactions between oxidative stress and ERS and that oxidative stress can cause ERS; meanwhile, ERS can also cause or aggravate oxidative stress [16, 17]. However, the mechanism of the interactions between oxidative stress and ERS is still not fully understood. Taken together, we will put forward a hypothesis that PERK-Nrf2 is likely to be the key crosstalk linked to oxidative stress and ERS.

ERS-initiated apoptotic signaling has been implicated in various chronic diseases including diabetes, cancer, and inflammation [18–20]. However, it has been not clear if ERS-initiated apoptosis is associated with the chronic lung injury caused by methamphetamine. Tertiary butylhydroquinone (TBHQ), an Nrf2 signaling pathway inducer, is demonstrated to induce remarkable antioxidant activity in a variety of cells and tissues [21]. On the basis of our hypothesis, the current study is designed to investigate if oxidative stress and ERS are involved in pulmonary toxicity and if PERK/Nrf2 is associated with the interaction between oxidative stress and ERS-initiated apoptosis in MA-induced pulmonary toxicity.

## 2. Materials and Methods

### 2.1. Chronic Methamphetamine Exposure

Forty-five male Wistar rats (200 ± 10 g) were purchased from Animal Resource Center, China Medical University (Certificate number: Liaoning SCSK 2012-0005). All of the 45 rats were randomly divided into control group, methamphetamine-treated group (MA), and methamphetamine plus TBHQ-treated group (MA + TBHQ). At the first week, intraperitoneal injection of methamphetamine with the dosage of 10 mg/kg for one week was administrated to the rats in MA group and MA + TBHQ group, and then increasing daily dosage of 1 mg/kg was administrated per week, until the sixth week, a daily dosage was increased to 15 mg/kg, respectively (twice per day for 6 weeks) [5, 22]. Meanwhile, the rats in control group were injected with an equal volume of 0.9% physiological saline solution. After administration of MA, rats in the MA + TBHQ groups then received intragastric administration of 12.5 mg/kg TBHQ [23]. Rats in the control and MA groups were intragastrically administered with an equal volume of 0.5% gum tragacanth. All animals were housed in a room with controlled temperature (18–22°C) and humidity (50%–70%) and were fed solid food and water ad libitum in an alternating 12 h light and 12 h dark cycle. All procedures followed the Guide for the Care and Use of Laboratory Animals of the National Institutes of Health (NIH), and all protocols were approved by the Institutional Animal Care and Use Committee of China Medical University.

### 2.2. Hemodynamic Measurements and Tissue Collection

After 6 weeks, rats in all groups were anaesthetized with 3% pentobarbital sodium (45 mg/kg, i.p.) prior to hemodynamic measurements, as described previously. Briefly, the right jugular vein was dissected and catheterized with a PV-1 catheter. The catheter was advanced via the right ventricle into the pulmonary artery for direct measurement of pulmonary arterial pressure (PAP). The right carotid artery was dissected and intubated with a PE-50 catheter to measure systemic blood pressure (SBP). Prior to catheterization, the PV-1 and PE-50 catheters were filled with saline containing 1% heparin. PAP and SBP were recorded using a polygraph system (RM6000; Nihon Kohden, Tokyo, Japan).

Of the 45 rats, 15 rats (five per group) were perfused with paraformaldehyde. The right lower lung tissues were dissected and then they were paraformaldehyde-fixed and paraffin-embedded. The lung sections (5 *μ*m thick) were prepared for hematoxylin-eosin (HE) staining, Eosin Van Gieson (EVG) staining, immunohistochemical (IHC) staining, immunofluorescence (IF), and TUNEL assay.

The other rats were then killed by overdose pentobarbital sodium. The rat lungs and pulmonary arteries were quickly dissected and removed on ice and stored at −80°C until further use for real-time PCR and western blotting.

### 2.3. Assessment of Remodeling of the Heart and Pulmonary Arteries

The hearts were dissected and taken out. Right ventricle (RV) and left ventricle plus interventricular septum (LV + S) were dissected and weighed separately to evaluate the extent of the right ventricular hypertrophy expressed as right ventricular index (RVI), which is calculated by weight ratio of the RV and LV + S.

Lung sections (5 *μ*m thick) were stained with hematoxylin and eosin (H&E), watched under light microscopy, and analyzed by Metamorphy/BX41 (UIC/OLYMPUS, USA/JAP). Vessels > 50 *μ*m were identified and measured at the 2 ends of the shortest external diameter of the distal PAs, and the percentage of medial wall thickness (medial wall thickness%) is calculated by ([2 × wall thickness/external diameter] × 100%).

Lung sections (5 *μ*m thick) were stained with EVG to localize collagen (staining red) and elastin (staining black brown) in lungs, watched under light microscopy, and analyzed by Metamorphy/BX41 (UIC/OLYMPUS, USA/JAP). The degree of muscularization was calculated as nonmuscularized (no evidence of any vessel wall muscularization), partially muscularized (smooth muscle cells (SMC) identifiable in less than three-quarters of the vessel circumference), or fully muscularized (SMC in more than three-quarters of the vessel circumference) to assess the remodeling of pulmonary arteries.

### 2.4. Immunohistochemical Staining

After processing the tissue and embedding in paraffin, 5 *μ*m thick sections were stained by immunohistochemical (IHC) procedures with Ultrasensitive TM S-P Kit and DAB Staining Kit (Maxin-Bio Co., China). IHC staining followed a basic indirect protocol using a citrate antigen retrieval method. Primary antibodies of mouse were anti-rat CHOP (1 : 100, Bioworld, USA) and goat anti-mouse *β*-actin (1 : 2000, Santa Cruz, California, USA) in TBS-T with 5% BSA overnight at 4°C. For the negative control, the primary antibody was replaced with 0.01 M phosphate-buffered saline (PBS) in the incubation step. The positive expression of these primary antibodies was examined and analyzed by BX51/Metamorphy (OLYMPUS/UIC, JAP/USA). At least, 6 medium and small pulmonary arteries were examined for each slide. For the convenience of understanding and statistical process, the content of protein in the pulmonary arteries was calculated as optical density average.

### 2.5. Immunofluorescence Staining

Tissue paraffin sections (5 *μ*m) were mounted on slides and placed in a 55°C oven for 10 min, deparaffinized in xylene (3x, 5 min), hydrated using an alcohol series, 100%, 95%, and 70% alcohol (each 3x, 5 min), and rinsed in water. The sections were processed for antigen retrieval by boiling the slides in 10 mM Citrate Buffer (pH 6.0). The slides were cooled at room temperature for 20 min, washed in PBS, and blocked in 10% normal serum overnight at 4°C. Immunofluorescence was performed on serial sections from each group using rabbit anti-bax and anti-bcl-2 antibodies (Proteintech, USA). The sections were incubated with primary antibodies for 1 h at room temperature. Subsequently, sections were stained with Alexa Fluor® 488 goat anti-rabbit secondary antibodies (Molecular Probes, Inc.). The fluorescent-stained sections were watched by Laser Confocal Scanning Microscope (FV1000S-SIM/IX81, OLYMPUS, JAP) and analyzed by Software Image J to calculate as the average intensity of the fluorescent signal. Six visual fields from each slide were imaged and quantified to assess the final mean fluorescent signal for each sample.

### 2.6. Western Blotting

Lung samples were homogenized in lysis buffer. Total protein from each sample was separated by sodium dodecyl sulfate polyacrylamide gel electrophoresis and transferred to PVDF membrane (Bio-Rad). The membranes were blocked by TBS-0.05% Tween-20 (TBS-T) with 5% nonfat dry milk for 60 min and were then incubated with mouse anti-rat NRF2 (1 : 500, Proteintech, USA), GCS (1 : 200, Beijing Biosynthesis Biotechnology Co., Ltd.), HO-1 (1 : 200, Beijing Biosynthesis Biotechnology Co., Ltd.), SOD 2 (Santa Cruz Biotechnology, Inc.), GRP 78 (1 : 2000, Proteintech, USA), PERK (1 : 1000, Bioworld, USA), p-PERK (1 : 200, Santa Cruz Biotechnology, Inc.), eIF2*α* (1 : 1000, Abcam, USA), p- eIF2*α* (1 : 1000, Cell Signaling, USA), ATF4 (1 : 1000, Protein tech, USA), CHOP (1 : 500, Biolworld, USA), bax (1 : 1000, Proteintech, USA), caspase 12 (1 : 1000, Proteintech, USA), and goat anti-mouse *β*-actin (1 : 2000, Santa Cruz, California, USA) antibodies in TBS-T with 5% BSA overnight at 4°C, respectively. A corresponding secondary antibody treatment is followed by enhanced chemiluminescence (ECL). The relative protein expression was quantified by densitometry using image quant software (Molecular Dynamics). The result of NRF2, GCS, HO-2, SOD 2, GRP78, p-PERK/PERK, p- eIF2*α*/eIF2*α*, ATF4, CHOP, bax, caspase 3, and caspase 12 was represented by the relative yield to the *β*-actin, respectively.

### 2.7. RNA Extraction and Quantitative Real-Time PCR

Total RNA was extracted with TRIzol Reagent (Invitrogen Co., USA) as described in its directions. The concentration and purification of total RNA were estimated with ultraspectrophotometer. The extracted RNA was reverse-transcribed with oligo dT primers and PrimeScript® RT reagent Kit Perfect Real Time (Takara Co. Japan). PCR experiments were done using the Chroma4 RT-PCR Detection System (Bio-Rad CFX96, CA, USA) and SYBR Green Supermix (Takara Co., Japan) according to the manufacturer's protocol. Sequences for gene specific primers corresponding to PCR targets were designed and synthesized by Lian-xing Bio-Tech Inc. The sequences for the Nrf2 and *β*-actin were described as Table 1.

### 2.8. TUNEL Assay

Terminal deoxynucleotidyl transferase-mediated dUTP-digoxigenin nick end labeling (TUNEL) TdT-mediated dUTP Nick-End Labeling was performed on lung sections according to the manufacturer's specifications (Beyotime Institute of Biotechnology, Shanghai, China). Addition of the chromogen diaminobenzidine tetra hydrochloride (DAB) resulted in a brown reaction product that was evaluated by light microscopy. The proportion of apoptosis was calculated as the number of apoptotic cells divided by the total number of cells. Six visual fields from each slide were imaged and quantified to assess the final mean percentage of apoptotic cells in the lungs.

### 2.9. Statistical Analysis

All data are expressed as the mean ± SD. Statistical comparisons were made by one-way analysis of variance, and statistical differences between two groups were established using the least significant difference test. Values of *P* < 0.05 were considered statistically significant.

## 3. Results

### 3.1. Comparison of Weight and Hemodynamic Indexes

The weight of rats was increased in all groups, but the percentage of weight changes was different between the groups after 6 weeks. The percentage of weight change was significantly lower in MA group than in control group (*P* < 0.01, versus control) (Table 2). MA has no significant effect on HR and SAP. In MA group, the PAP and RVI were significantly increased, but they can be reversed by TBHQ (Table 3).

### 3.2. Effect of TBHQ on MA-Induced Pulmonary Vascular Remodeling

**Figure 1:**
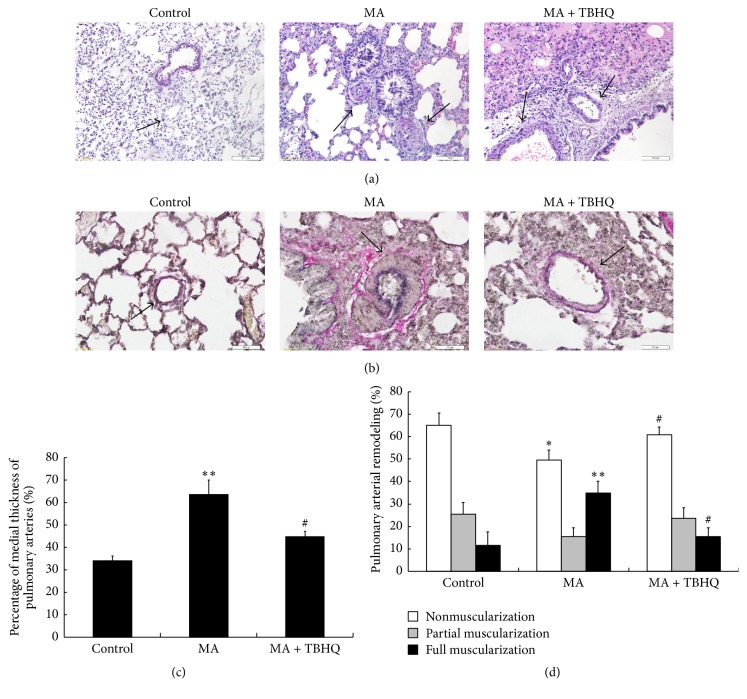
Effect of TBHQ on MA-induced pulmonary vascular remodeling. (a) Hypertrophy of pulmonary vessel wall by H&E staining (Olympus BX 51, Japan, ×400); (b) the muscularization of pulmonary arteries by EVG staining; (c) the percentage of medial wall thickness in different groups (Olympus BX 51, Japan, ×400); (d) muscularization degree of pulmonary arteries in different groups. The changes of pulmonary arteries were marked by black arrows. Compared with the control group, in MA group, the lumen was significantly narrowed and the wall of pulmonary arteries was markedly thickened, which were attenuated by TBHQ. Values are expressed as mean ± SD (*n* = 5). ^*∗*^*P* < 0.05, ^*∗∗*^*P* < 0.01, compared with the control group; ^#^*P* < 0.05, compared with the MA group. MA: methamphetamine; THBQ: tertiary butylhydroquinone.

Hypertrophy of pulmonary vessel wall was evaluated as the percentage of medial wall thickness (medial wall thickness%) by H&E staining (Figure 1(a)). The percentage of medial wall thickness was increased from 34.1% ± 2.1% in control group to 64.5% ± 6.4% in MA group and was markedly inhibited in MA + TBHQ at 44.7% ± 2.3% (Figure 1(c)). The muscularization of pulmonary arteries was detected by EVG staining and was investigated under light microscopy (Figure 1(b)). Elastin was stained in black, and collagen was stained in red. The rates of nonmuscularization, partial muscularization, and full muscularization in the control group were 64.9, 25.5, and 11.6%, respectively; in the MA group, these values were 49.5, 15.5, and 35%, respectively; and, in the MA + TBHQ group, these values were 60.9, 23.6, and 15.5%, respectively (Figure 1(d)). These results indicate that MA significantly promotes pulmonary vascular remodeling, whereas TBHQ attenuates the effects of MA.

### 3.3. Effect of MA and TBHQ on Nrf2-Mediated Antioxidative Stress in Lungs

**Figure 2:**
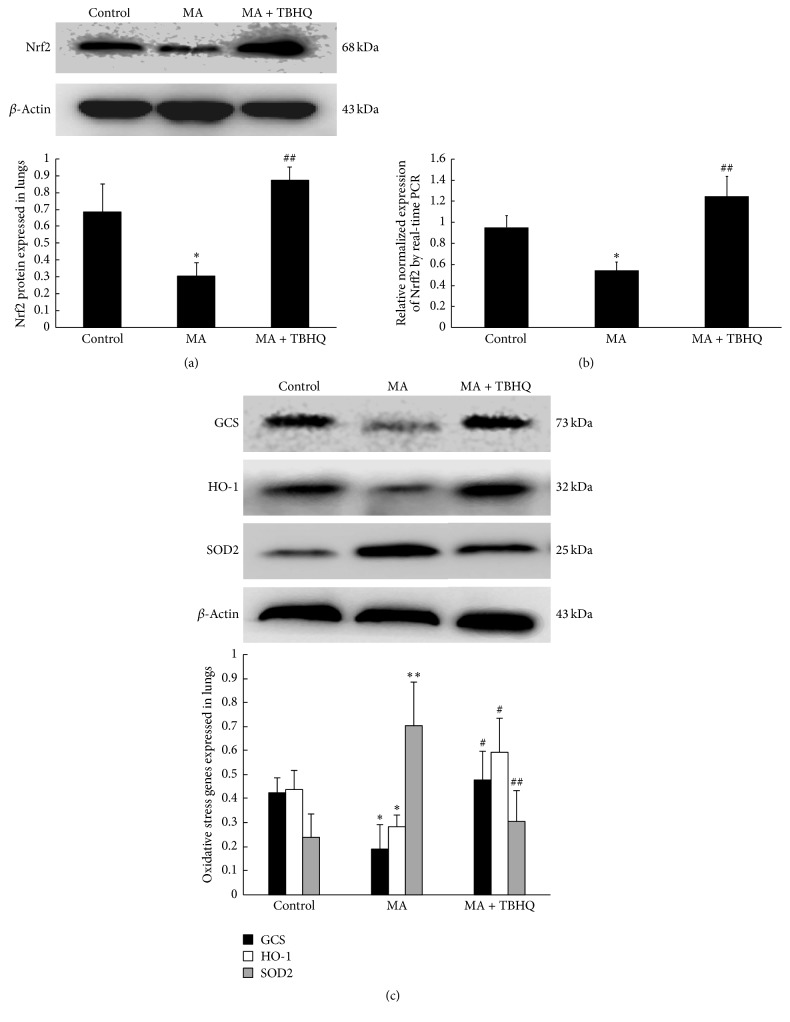
Effect of MA and TBHQ on Nrf2-mediated antioxidative stress in lungs. (a) Nrf2 protein expression in lungs; (b) Nrf2 level in different groups by real-time PCR; (c) oxidative genes expression in different groups. Values are expressed as mean ± SD (*n* = 5). ^*∗*^*P* < 0.05, ^*∗∗*^*P* < 0.01, compared with the control group; ^#^*P* < 0.05, ^##^*P* < 0.01, compared with the MA group. MA: methamphetamine; THBQ: tertiary butylhydroquinone.

Protein extracts were subjected to western blot. Compared with control group, MA significantly downregulated the Nrf2 expression, which was markedly upregulated by TBHQ (Figure 2(a)). Additionally, the results of Nrf2 by real-time PCR were coincided with the western blot (Figure 2(b)). And as shown in Figure 2(c), the antioxidative enzymes GCS and HO-1 were decreased, but oxidative enzyme SOD2 was increased by MA, which was reversed by TBHQ. These results indicated that MA impeded Nrf2-medicated antioxidative stress and impaired the ability of antioxidative stress, which aggravated the oxidative stress.

### 3.4. Effect of TBHQ on the Lasting-ERS Chronically Exposed to MA

**Figure 3:**
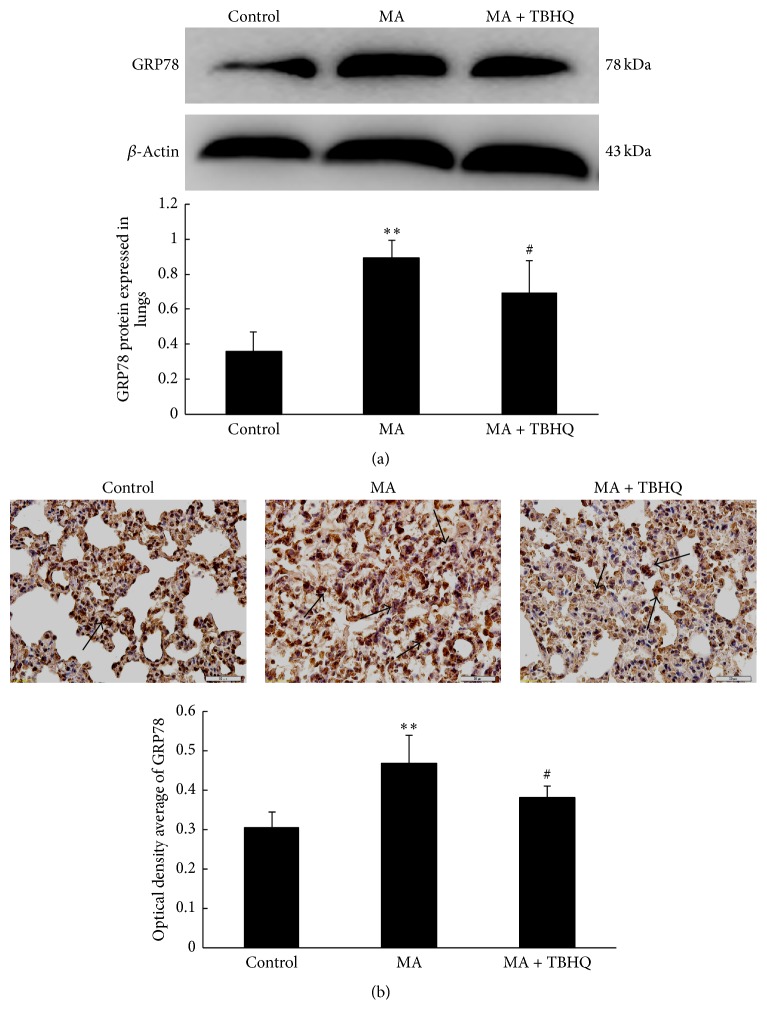
Effect of TBHQ on the lasting ERS chronically exposed to MA. (a) GRP78 expression in rat lungs by western blot; (b) GRP78 protein expression illustrated by immunohistochemical analysis (×400). Cross sections were stained with rabbit anti-GRP78 (brown) and counterstained with hematoxylin (blue) in different groups. Values are expressed as mean ± SD (*n* = 5). ^*∗∗*^*P* < 0.01, compared with the control group; ^#^*P* < 0.05, compared with the MA group. MA: methamphetamine; THBQ: tertiary butylhydroquinone.

GRP78 is the key signal of the ERS. The western blot assay demonstrated that the expression of the GRP78 is higher in MA group than in the control group (Figure 3(a)). Additionally, immunohistochemistry with anti-GRP78 antibody revealed strong GRP78 expression in rat lungs, compared with the control group (Figure 3(b)). However, TBHQ can obviously downregulate the expression of GRP78 in rat lungs. These suggested that the prolonged stimulation excessively activated ERS, which can be alleviated by TBHQ.

### 3.5. Effect of MA and TBHQ on the PERK Signaling Pathway

**Figure 4:**
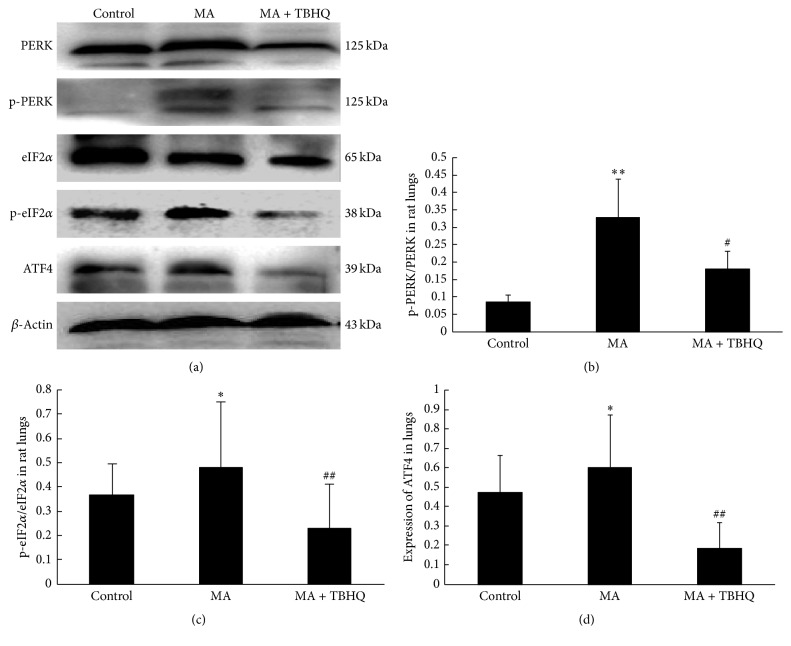
Effect of MA and TBHQ on the PERK signaling pathway. (a) PERK signaling expression by western blot; (b) p-PERK/PERK in lungs in different groups; (c) p-eIF2*α*/eIF2*α* in different groups; (d) ATF4 protein expression in different groups. Values are expressed as mean ± SD (*n* = 5). ^*∗*^*P* < 0.05, ^*∗∗*^*P* < 0.01, compared with the control group; ^#^*P* < 0.05, ^##^*P* < 0.01, compared with the MA group. MA: methamphetamine; THBQ: tertiary butylhydroquinone.

Results from western blot analysis (Figure 4(a)) demonstrated that the expressions of PERK, p-PERK, and ratio of p-PERK/PERK were all significantly upregulated in lungs in MA group, compared with control group, and that they were decreased in MA + TBHQ group, compared with MA group (Figure 4(b)). PERK signaling was activated to induce rapid phosphorylation of eIF2*α*. Western blot was carried out to further demonstrate that, in MA group, signal eIF2*α* was phosphorylated compared with control group. And p-eIF2*α* expression was decreased after administration of TBHQ (Figure 4(c)). Downstream signal ATF4 expression is also reversed by TBHQ from MA (Figure 4(d)). These results indicated that chronic exposure to MA induced lasting ERS by excessively phosphorylating PERK/eIF2*α* signaling.

### 3.6. Effect of MA and TBHQ on ERS-Initiated Apoptosis

**Figure 5:**
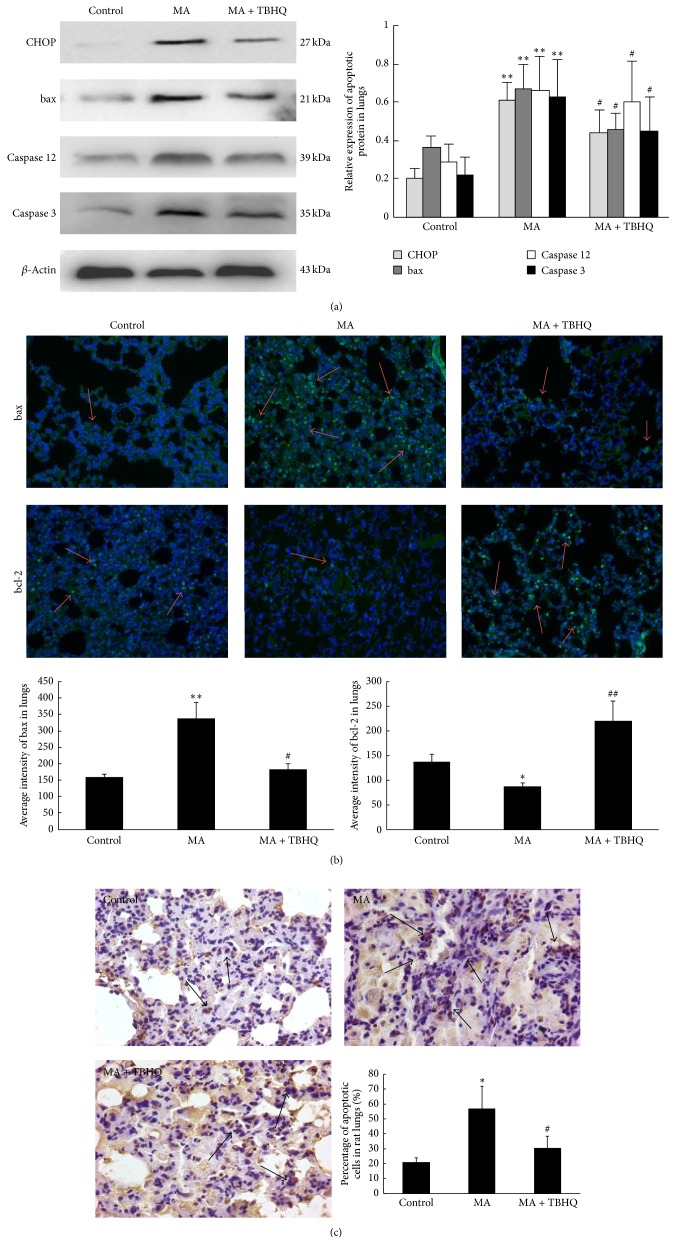
Effect of MA and TBHQ on ERS-initiated apoptosis. (a) Apoptotic cytokines CHOP, bax, caspase 12 and caspase 3 expressed in different groups by western blot; (b) immunofluorescence assay for bax and bcl-2 in different groups (×400). Higher positive expression (green) of bax in MA group than in control group, but it is contrary to the expression of bcl-2, which can be reversed by TBHQ. (c) TUNEL assay in different groups (×400). TUNEL-positive staining is indicated by dark brown particles in cell nucleus. The proportion of apoptosis significantly increased in the MA group, compared with control group. Yet it decreased in the MA + TBHQ group. Values are expressed as mean ± SD (*n* = 5). ^*∗*^*P* < 0.05, ^*∗∗*^*P* < 0.01, compared with the control group; ^#^*P* < 0.05, ^##^*P* < 0.01, compared with the MA group. MA: methamphetamine; THBQ: tertiary butylhydroquinone.

Results from western blot analysis demonstrate that compared with control group, CHOP, bax, caspase 3, and caspase 12 protein expressions were significantly increased in MA groups, which were markedly decreased after administration of TBHQ (Figure 5(a)). The immunofluorescence staining results (Figure 5(b)) showed that there is higher positive expression (green) of apoptotic cytokine bax in MA group than in control group, and it is ameliorated by TBHQ. However, it is contrary to the expression of anti-apoptotic cytokine bcl-2, which implied that TBHQ can ameliorate the ERS-initiated apoptosis by MA. TUNEL-positive staining is indicated by dark brown. TUNEL-positive cells are infrequently observed in the control group. The proportion of apoptosis significantly increased in the MA group (*P* < 0.05 versus control). Yet it decreased in the MA + TBHQ group (*P* < 0.05 versus MCT) (Figure 5(c)).

## 4. Discussion

Results from the present study showed that chronic exposure to methamphetamine reduced weight growth of rats and induced pulmonary toxicity of rats by increasing the pulmonary arterial pressure, promoting the hypertrophy of right ventricle and the remodeling of pulmonary arteries. MA inhibited the Nrf2-mediated antioxidative stress by downregulation of Nrf2, GCS and HO-1, and upregulation of SOD2. MA damaged the ability of anti-oxidative stress to aggravate oxidative stress. Overexpression and phosphorylation of PERK rapidly phosphorylated eIF2*α* and activated the PERK/eIF2*α*/ATF4 signaling. This chronic stimulation caused lasting ERS and further induced apoptosis by the increase in CHOP, bax, caspase 3, caspase 12 and decrease in bcl-2. These changes can be reversed by antioxidant TBHQ through upregulating expression of Nrf2. The above results indicated that TBHQ can alleviate MA-induced oxidative stress which can accelerate ERS to initiate PERK-dependent apoptosis and that PERK/Nrf2 is likely to be the key crosstalk between oxidative stress and ERS in MA-induced chronic pulmonary toxicity.

As a powerful addictive drug, MA leads to multiple organs damage, such as brain, heart, and lung [24–26]. The redox imbalances and generation of free radicals such as ROS can lead to oxidative stress [27]. Oxidative stress is one of the reasons of MA-induced neurotoxicity [28]. In this study, it is found that, in rat lungs, the antioxidative enzymes GCS and HO-1 were decreased, but oxidative enzyme SOD2 was increased by MA, which was reversed by TBHQ. These results are in accord with the previous reports that the damage in nervous system caused by MA can be attenuated by antioxidants [29, 30]. Therefore, TBHQ attenuated MA-induced pulmonary toxicity by increasing and activating Nrf2 to strengthen the ability of antioxidative stress.

Oxidative stress can disrupt the function of endoplasmic reticulum (ER) and lead to endoplasmic reticulum stress (ERS) [31]. To survive the ERS, the three mechanisms, unfolded protein response (UPR), the ER-overload response (EOR), and the ER-associated degradation (ERAD), will be activated [28]. UPR is vital to ensure the ER function and cell survival [32]. GRP78 is one of the most highly expressed ER resident chaperones in the condition of UPR which belongs to heat-shock protein (Hsp70) family [32, 33]. When the unfolded/misfolded proteins accumulate in the ER, the three ER transmembrane receptors will release GRP78 and activate and initiate signaling cascades designed to protect the cells or lead to apoptosis [34, 35]. GRP78 is the sign of UPR and ERS [36]. In our study, MA induced the high expression of GRP78, which indicated that ERS was involved in chronic lung injury. The GRP78 in MA + TBHQ group was less than MA group. It demonstrated that TBHQ alleviated the lasting ERS to some extent.

ER transmembrane sensors include PERK, ATF6, and IRE1 [8]. PERK is the one of three transmembrane proteins of the UPR signaling and activated by the ERS [37, 38]. The phosphorylation of PERK and its downstream factor eIF2*α* attenuate the synthesis of protein and restore ER homeostasis [39, 40]. The Nrf2 is known as a substrate of PERK [41]. Phosphorylation of PERK can cause a conformational change of Nrf2 protein by triggering the dissociation of Keap1-Nrf2 complex, and the dissociation of Nrf2 into the nucleus upregulated expression of antioxidant genes [42]. Therefore, oxidative stress and endoplasmic reticulum stress has some certain relevance through PERK/Nrf2 pathway, which is why we choose PERK-dependent pathway of ERS. In our study, it is found that MA inhibited the expression of Nrf2 in lungs to phosphorylate PERK/eIF2*α* and activate ATF4/CHOP-dependent apoptosis. Hence, interfering with Nrf2 response caused accumulation of damaged proteins within the ER, leading to PERK-dependent apoptosis [43]. It is a hint that oxidative stress induced endoplasmic reticulum stress, and excessive endoplasmic reticulum stress can also cause or aggravate oxidative stress due to PERK/Nrf2 crosstalk [41, 44].

A rich body of evidence has demonstrated that TBHQ can activate antioxidant responsive element (ARE) and upregulate Nrf2 expression to attenuate the damage of nervous system [45, 46]. The antioxidant TBHQ is effective in protecting against cellular dysfunction induced by oxidative stress inducers in various cell types [47]. In our study, it is found that, in rat lungs, TBHQ not only upregulated the level of Nrf2 and its related antioxidative genes but also inhibited the expression of GRP78 and abrogated the PERK/eIF2*α*/ATF4/CHOP-dependent apoptosis. Taken together with the above results, it is indicated that TBHQ attenuated ER stress to abrogate ERS-initiated apoptosis by activating antioxidative stress in MA-induced chronic pulmonary toxicity. It hinted that the lasting endoplasmic reticulum stress-apoptosis can be alleviated by the adjusting the balance between oxidation and reduction [42, 48].

In summary, both oxidative stress and ERS may be involved in MA-induced pulmonary toxicity, and oxidative stress can accelerate ERS to further initiate PERK-dependent apoptosis, which can be alleviated by the antioxidant TBHQ. These results from our study implied that PERK/Nrf2 is likely to be the key crosstalk between oxidative stress and ERS in MA-induced chronic pulmonary toxicity.

## Figures and Tables

**Table 1:** 

Accession number	Species	Primer	Primer sequences (5′ to 3′)
RA016146	Rat	(Nrf2) -Forward	AGAGATGGAACTGACTTGGCAAGAG
RA016146	Rat	(Nrf2) -Reversed	TGCATCTGGATGAATTGAACAGG
RA015375	Rat	(*β*-actin) -Forward	GGAGATTACTGCCCTGGCTCCTA
RA015375	Rat	(*β*-actin) -Reversed	GACTCATCGTACTCCTGCTTGCTG

**Table 2:** Comparison of the rat weights in different groups.

	Preweight (g)	Postweight (g)	Percentage of weight change (%)
Control (*n* = 15)	198.7 ± 9.8	299.4 ± 18.4	50.8 ± 8.8
MA (*n* = 10)	203.6 ± 5.8	232.6 ± 12.1^*∗∗*^	14.3 ± 6.0^*∗∗*^
MA + TBHQ (*n* = 12)	199.4 ± 7.7	250.0 ± 11.9^*∗∗*,#^	25.4 ± 5.9^*∗∗*,##^

Data are means ± SD. ^*∗∗*^*P* < 0.01, versus control group. ^#^*P* < 0.05, ^##^*P* < 0.01, versus MA group. MA, methamphetamine. TBHQ, tertiary butylhydroquinone.

**Table 3:** Comparison of HR, mSAP, mPAP, and RVI in different groups.

	HR (bmp)	mSAP (mmHg)	mPAP (mmHg)	RVI
Control (*n* = 15)	375 ± 31	138.9 ± 8.5	15.7 ± 1.9	0.23 ± 0.02
MA (*n* = 10)	376 ± 39	138.1 ± 16.7	20.5 ± 2.0^*∗*^	0.37 ± 0.03^*∗∗*^
MA + TBHQ (*n* = 12)	378 ± 37	139.4 ± 17.3	17.4 ± 2.4^#^	0.27 ± 0.04^##^

Data are means ± SD; ^*∗*^*P* < 0.05, ^*∗∗*^*P* < 0.01, versus control group. ^#^*P* < 0.05, ^##^*P* < 0.01, versus MA group. HR, heart rate; mSAP, mean systemic arterial pressure; mPAP, mean pulmonary arterial pressure; RVI, right ventricular index; MA, methamphetamine; TBHQ, tertiary butylhydroquinone.
